# Supplementary material for: Host Metabolic Changes during Mycobacterium Tuberculosis Infection Cause Insulin Resistance in Adult Mice
Source: J Clin Med. 2022 Mar 16;11(6):1646. doi: 10.3390/jcm11061646 (PMC8948975; doi:10.3390/jcm11061646)
Supplement: Supplementary file 1 [file jcm-11-01646-s001.zip › jcm-1605781-supplementary.pdf]

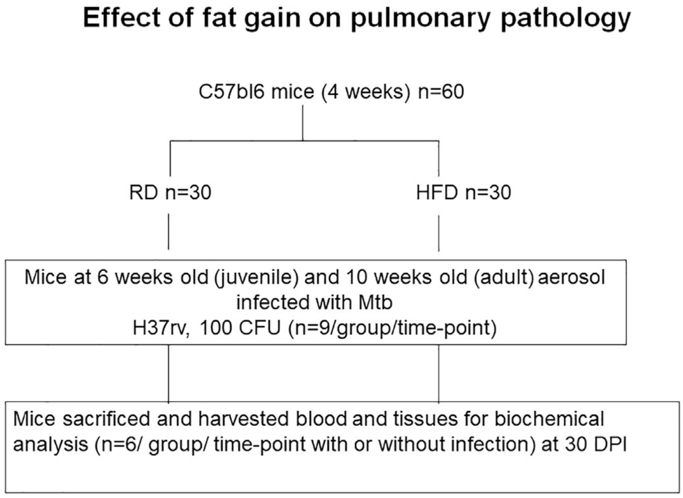

Figure S1. Flowchart of experimental design.

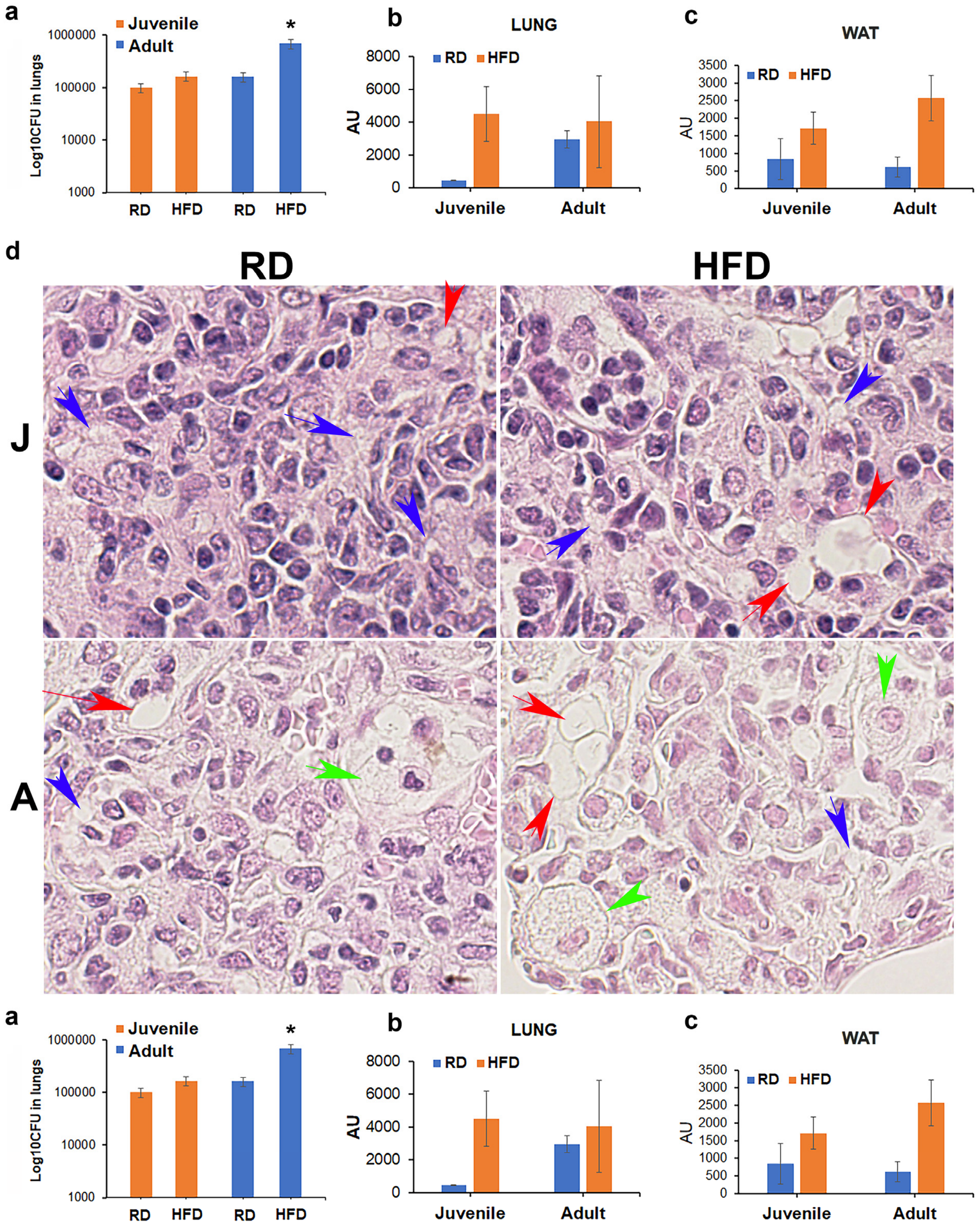

Figure S2. (a) Number of Log_{10}CFU in the lungs of *Mtb* infected RD and HFD mice. Bacterial counts were determined from lung homogenates plated on agar medium. The values plotted are mean±SE from n=5 animals per group. The error bars represent standard error of the mean. *, P<0.05 compared to adult infected RD mice. Quantification of the A-R staining of *Mtb* in the (b) lungs and (c) WAT of infected juvenile and adult mice using Image J software. (d) H&E sections of the lungs demonstrated the presence of foamy macrophages (green arrow) and accumulation of micro-lipids (blue arrow) and macro-lipids (red arrow) in the lungs (x40 magnified).

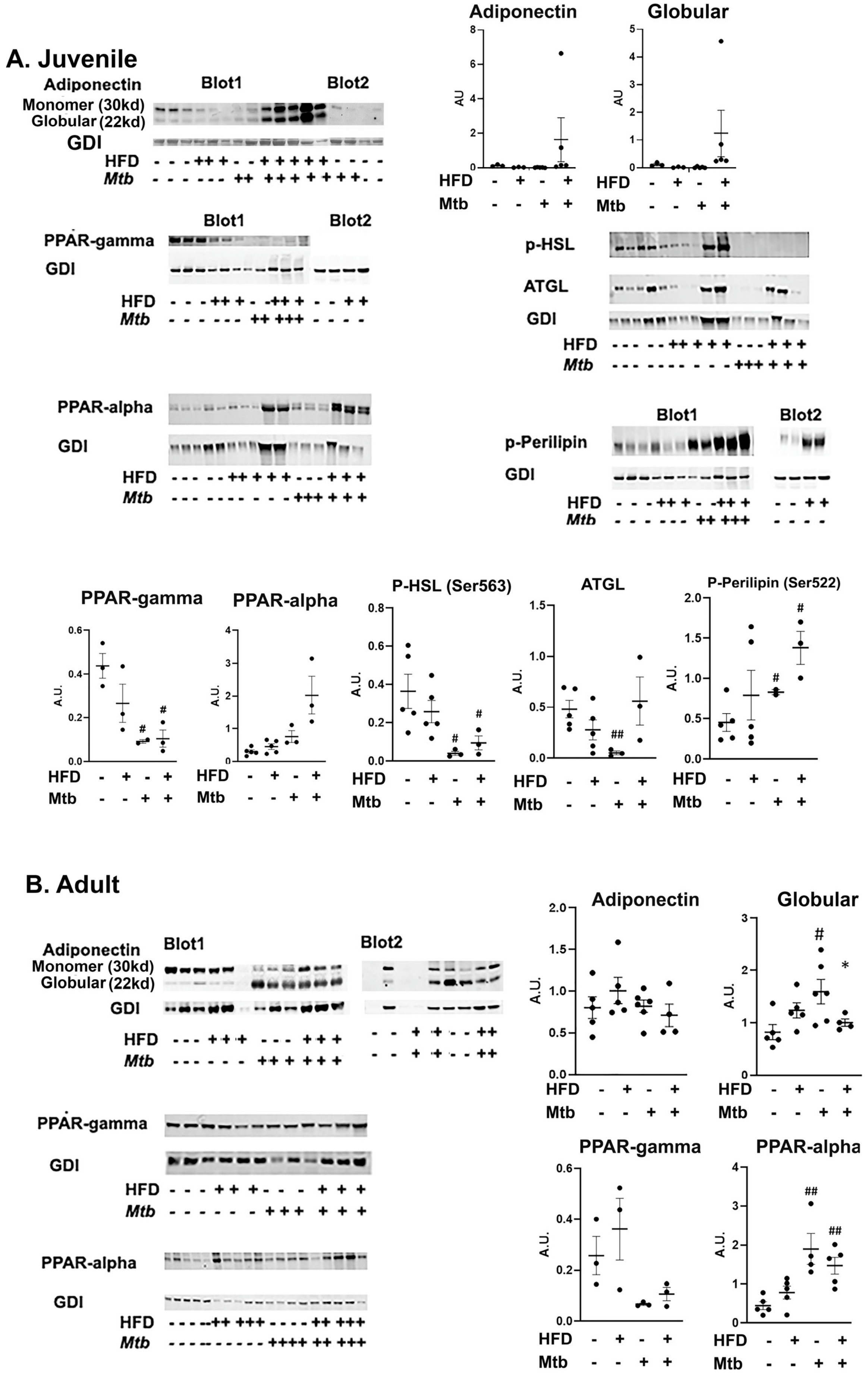
A. Juvenile
Adiponectin
Blot1
Blot2
Monomer (30kd)
Globular (22kd)
GDI
HFD
Mtb
Adiponectin
Globular
AU
PPAR-gamma
PPAR-alpha
p-HSL
ATGL
p-Perilipin
P-HSL (Ser563)
P-Perilipin (Ser522)
A.U.
B. Adult

Figure S3. (a) Immunoblot analysis of the levels of adipogenic markers (adiponectin monomer, adiponectin globular, PPARγ), lipases (P-HSL, P-Perilipin), lipid oxidation marker (PPARα) in WAT lysates of infected and uninfected juvenile mice. (b) Immunoblot analysis of the levels of adipogenic markers (adiponectin monomer and adiponectin globular), lipases (P-HSL, P-Perilipin), PPARα, PPARγ in WAT lysates of infected and uninfected adult mice. GDI was used as loading control. Fold changes in the protein levels were normalized to GDI expression and are represented as a dot plot. The error bars represent standard error of the mean. #, P<0.05, ##, P<0.01, compared to uninfected RD mice, *, p<0.005, compared to infected RD mice (n=5/group).

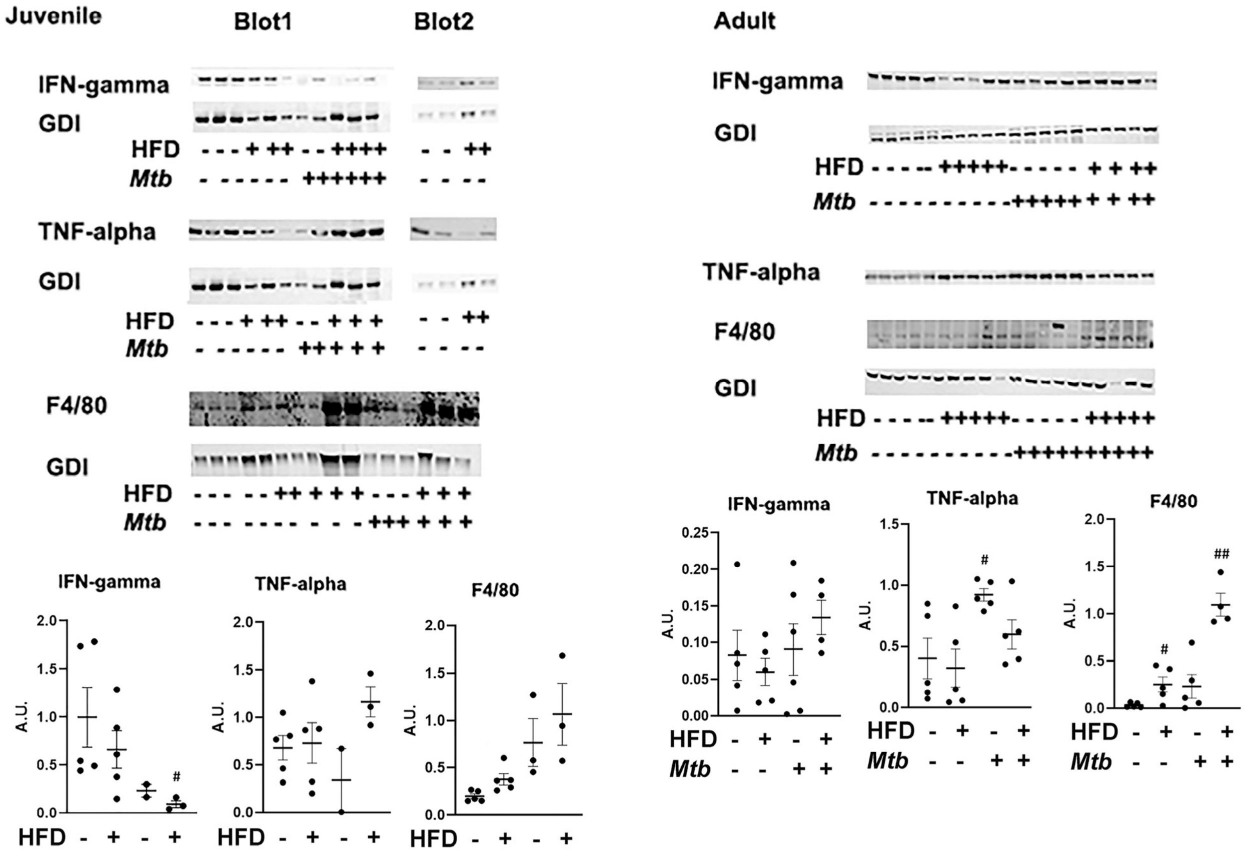

Figure S4. Immunoblot analysis of the levels of inflammatory markers (IFNγ, TNFα) and macrophage marker (F4/80) in WAT lysates of infected and uninfected juvenile and adult mice. GDI was used as loading control. Fold changes in the protein levels were normalized to GDI expression and are represented as a dot plot. The error bars represent standard error of the mean. #, P<0.05 and ##, P<0.01, compared to uninfected RD mice (n=5/group).

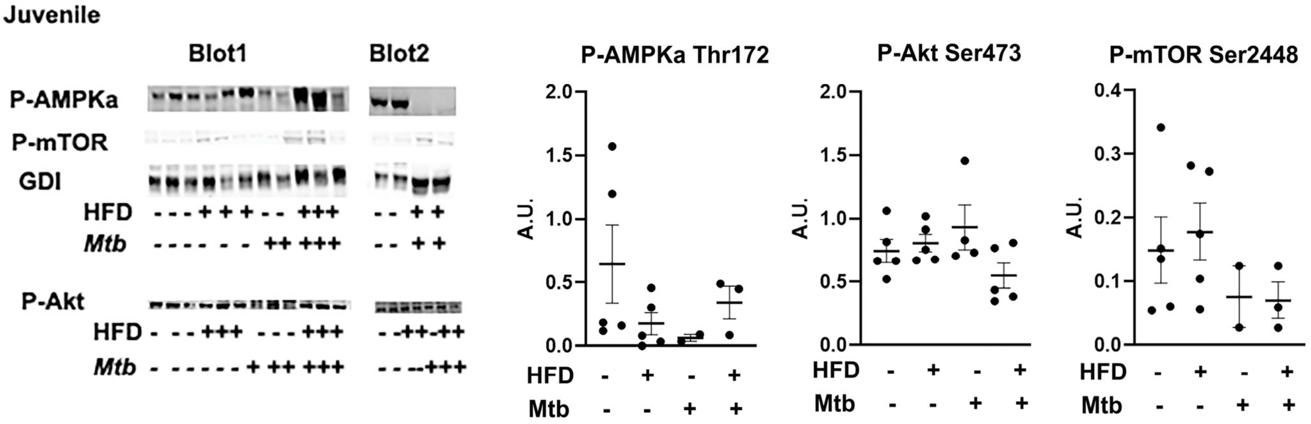

Figure S5. Immunoblot analysis of cell metabolic pathway proteins (P-AMPKα Thr 172, P-Akt Ser 473, P-mTOR Ser2448) in the WAT lysates of infected and uninfected juvenile mice. GDI was used as loading control. Fold changes in the protein levels were normalized to GDI expression and are represented as a dot plot. The error bars represent standard error of the mean.
